# SCAI Staging Application for Acute Myocardial Infarction-Related Cardiogenic Shock at a Single-Center Russian Registry

**DOI:** 10.3390/jcm12247739

**Published:** 2023-12-17

**Authors:** Vyacheslav V. Ryabov, Oleg O. Panteleev, Maria A. Kercheva, Alexei A. Gorokhovsky, Anna G. Syrkina, Natalia Y. Margolis

**Affiliations:** 1 Cardiology Research Institute, Tomsk National Research Medical Center, Russian Academy of Sciences, 634012 Tomsk, Russiapanteleevoo@cardio-tomsk.ru (O.O.P.); sag@cardio-tomsk.ru (A.G.S.);; 2 Cardiology Division, Siberian State Medical University, 2 Moscovsky Trakt, 634055 Tomsk, Russia

**Keywords:** cardiogenic shock, myocardial infarction, SCAI, acute coronary syndrome

## Abstract

Aim: To access the features of the course of myocardial infarction (MI) in patients with different stages of MI complicated by cardiogenic shock (MI CS) according to the SCAI scale. Methods: We retrospectively described the portrait of CS MI (*n* = 117) at different stages of SCAI from the hospital MI registry (*n* = 1253). Results: Hospital mortality increased from stage to stage (*p* ≤ 0.001). Significant differences in biochemical parameters were found both for indicators characterizing intensive care measures, such as the presence of mechanical lung ventilation or an intra-aortic balloon pump, and for indicators of organ hypoperfusion such as lactate level, pHv (7.39 (7.36; 7.44) at stage A–B; 7.14 (7.06; 7.18) at stage E), creatinine, and glomerular filtration rate. Parameters related to MI characteristics, such as instrumental and laboratory data, anamnesis of ischemia, and performed treatment, did not differ between groups. Polynomial logistic regression showed that lactate level, mechanical ventilation, and monocyte count upon admission (1.15 (0.96; 1.23) at stage A–B; 0.78 (0.49; 0.94) at stage E, *p* = 0.005) correlated with CS severity. Conclusion: The characteristics of MI at different stages of SCAI do not have differences and do not determine the severity of shock. We revealed a high discriminatory potential of the pH level in predicting refractory shock. The value of monocytes at admission may be a promising predictor of the severity of MI CS. The question of the causes of heterogeneity of MI CS, taking into account the homogeneity of MI characteristics, remains open and promising.

## 1. Introduction

Myocardial infarction complicated by cardiogenic shock (MI CS) is generally known to be caused by injury involving 40% or more of myocardial mass [1]. As a result, the pumping function of the heart is significantly inhibited, which leads to reduced cardiac output and organ hypoperfusion [2]. The effectiveness of early myocardial revascularization and active use of modern therapy aimed at maintaining the pumping function of the heart is convincingly supported by the available data; nevertheless, in-hospital mortality in MI CS patients remains extremely high [3,4,5,6,7,8,9,10,11]. The prevention, course, and prognosis of CS show no positive trends, which may be due to the heterogeneity of the study group, complicated early CS diagnosis, or the lack of timely and adequate impact on the key pathogenetic mechanisms of its development and progression [12,13].

Until recently, the lack of a unified classification complicated the study of the phenotypic heterogeneity of CS [14,15,16,17,18,19,20]. In 2019, the Society for Cardiovascular Angiography and Intervention (SCAI) presented a new CS classification system that demonstrated a strong association between shock stages and mortality in a heterogeneous patient population [21,22,23]. However, the original and updated versions of the SCAI shock classification lacked specific reference values for the key parameters used to define hypotension, hypoperfusion, and treatment intensity, which has caused some differences between studies. Widespread use of the SCAI shock classification requires objective CS staging to easily apply it in real clinical practice. Thus, the SCAI shock staging requires the determination of the specific variables that primarily affect shock severity. The SCAI scale does not particularly provide parameters used to assess the severity of acute MI. It remains unknown whether they contribute to staging and whether they can be used as markers to determine shock severity. Our aim was to access the features of the course of MI in patients with different stages of MI CS according to the SCAI scale. 

## 2. Materials and Methods

### 2.1. Study Design

**Figure 1:**
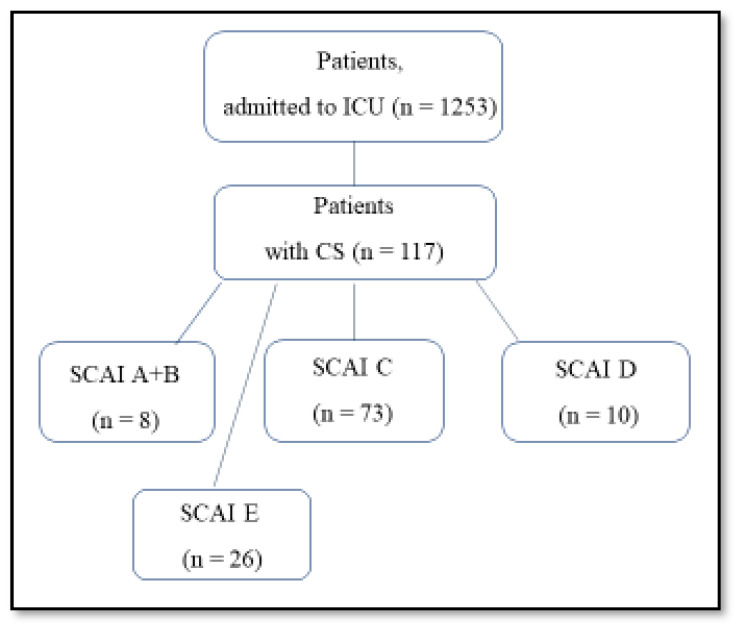
Design of the study. Footnotes: Abbreviations: CS, cardiogenic shock; ICU, intensive care unit.

In our retrospective registry study, we analyzed 1253 medical records of patients from the MI registry of the Cardiology Research Institute, Tomsk National Research Medical Center, for the period from 1 January 2020 to 12 December 2020, with 117 of these having a CS diagnosis according to international classification of deposits (ICD)-10 upon admission; admission diagnoses were defined as all ICD-10 codes registered within 1 day of admission to the intensive care unit (ICU) (Figure 1).

### 2.2. Clinical, Laboratory, and Instrumental Data

The data from medical records were used to generate a database with demographic data, vital signs (upon admission and when the condition worsened), clinical and laboratory data, details of the procedures and the treatment performed, MI temporal characteristics, and outcomes (315 different parameters in total). The vital signs, clinical measurements, and laboratory parameters obtained upon admission were defined as the primary values recorded on admission to the ICU, or as the values recorded closest to admission. Patients were retrospectively assigned to one of the updated SCAI shock classification groups (A = at risk; B = hypotension; C = hypoperfusion; D = deterioration; E = extreme) (Table 1).

### 2.3. Statistical Analysis

Categorical indicators were presented as absolute (*n*) and relative (%) frequencies. Quantitative indicators were presented as median (Me) and interquartile range (Q1; Q3). Either Pearson’s χ^2^ test or Fisher’s exact test was used to compare categorical scores between independent groups. To compare quantitative indicators in three or more independent groups, the Kruskal–Wallis test was used, with post hoc comparisons using the Mann–Whitney test and Bonferroni corrections for multiple comparisons. The critical level of significance when testing statistical hypotheses was 0.05. A multinomial logistic regression model was constructed to identify predictors of different stages of SCAI. The SCAI = A and SCAI = B groups were combined due to their small size. Statistical analysis was performed using Jamovi 2.3.13.

## 3. Results

### 3.1. Clinical Data

Patients were divided into stages as follows: A, 2% (2); B, 5% (6); C, 62.4% (73); D, 8.5% (10); E, 22.2% (26) (Table 2).

### 3.2. Comparison of Patient Characteristics Depending on the CS stage Classification on the SCAI Scale

Patients in SCAI shock stages C, D, and E were significantly older: A and B: 65.5 (58.3; 75.5); C: 73 (66; 81); D: 80.5 (73.3; 82); and E: 80 (78.3; 86.5) (*p* = 0.009). Stages A and B were represented only by men, while stages C, D, and E equally involved men and women (53.4%; 50%; 69.2% for women, respectively). In-hospital mortality for the groups was as follows: A and B: 37.5% (3); C: 43.8% (32); D: 60% (6); E: 88.5%, and these numbers increased significantly from stage to stage (*p ≤* 0.001). Among objective status data, the groups were found to differ in systolic blood pressure and mean blood pressure levels, as well as Glasgow coma scale. Significant differences in biochemical parameters were found for indicators of organ hypoperfusion: lactate level, pHv, creatinine, and glomerular filtration rate. On average, significant differences in the parameters were found for indicators characterizing intensive care measures, such as the presence of mechanical lung ventilation or an intra-aortic balloon pump. Statistically significant differences were not revealed during the assessment of the parameters related to MI characteristics: electrocardiography morphology (MI with ST segment elevation, MI without ST segment elevation, Q-forming MI), the level of cardiac enzymes (creatine phosphokinase (CK), CK-MB, troponin I), echocardiographic characteristics, coronary angiography data (single-, double-, triple-vessel coronary artery disease), previous coronary interventions (percutaneous coronary intervention (PCI), coronary artery bypass graft (CABG)), localization, performed treatment (PCI, thrombolytic therapy (TLT), pharmacoinvasive, conservative), time indicators (onset of symptoms to hospital admission and door-to-balloon time), and cardiovascular history (primary, recurrent MI).

### 3.3. Associations between MI Characteristics and SCAI Shock Stage

The association between independent (predictor) variables and the SCAI shock stage was assessed using a developed polynomial logistic regression model (Table 3).

**Figure 2:**
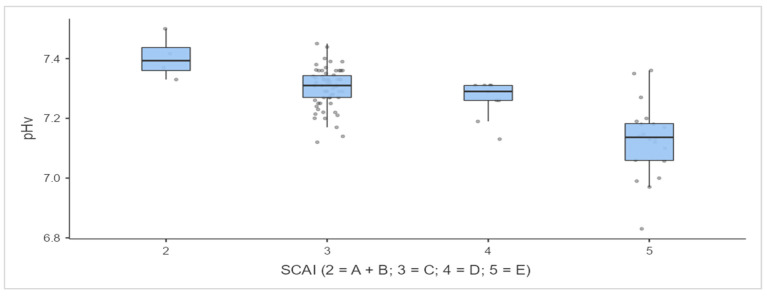
Level of pHv at admission. Footnotes. A—at risk; B—beginning (hypotension without hypoperfusion); C—classic (hypoperfusion without deterioration); D—deteriorating (hypoperfusion with deterioration); E—extremis (hypoperfusion with deterioration and refractory shock).

**Figure 3:**
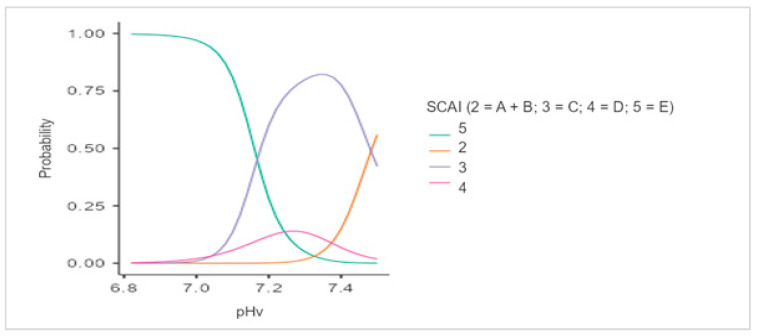
Dependence of the probability of classification into a group with a given level of SCAI on the average values of pHv. Footnotes: A = at risk; B = beginning (hypotension without hypoperfusion); C = classic (hypoperfusion without deterioration); D = deteriorating (hypoperfusion with deterioration); E = extremis (hypoperfusion with deterioration and refractory shock).

The model demonstrated good model quality metrics: a high pseudo-determination coefficient (Nagelkerke R.N.), a significance level of the model of *p* = 1.14 × 10^–12^, an insignificant spread of predicted values, and a satisfactory value for the Akaike (AIC) information criterion. All the indicators, except for spO_2_/FioO_2_, can be considered critical in assessing the risk of MI CS, particularly pHv (Figure 2 and Figure 3). 

**Figure 4:**
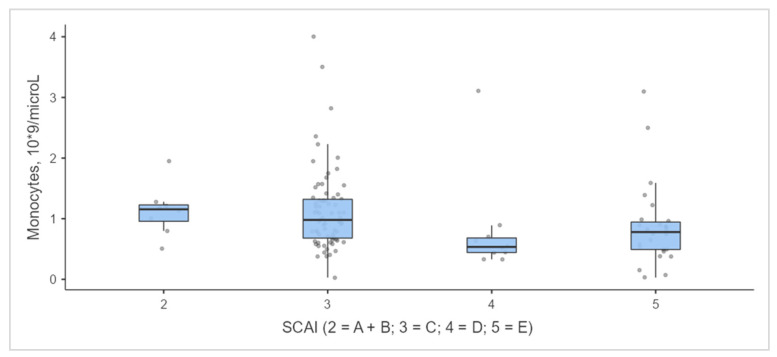
Levels of monocytes at admission. Footnotes: A—at risk; B—beginning (hypotension without hypoperfusion); C—classic (hypoperfusion without deterioration); D—deteriorating (hypoperfusion with deterioration); E—extremis (hypoperfusion with deterioration and refractory shock).

Interestingly, along with such indicators as lactate level and mechanical ventilation, which have previously been used in CS prognostic scales [7,15], the monocyte count upon admission in patients in the SCAI shock stages from 3 to 5 correlated with shock severity (Figure 4, Table 3).

## 4. Discussion

The SCAI shock stage was an independent predictor of adverse outcome in all clinical subgroups, regardless of shock etiology (MI, decompensated heart failure, etc.) [23]. Therefore, this classification can be used as a tool to identify possible causes of high mortality from MI CS.

Most of the studies on SCAI shock classification have included a mixed patient population. In the largest study, by Jentzer et al. (*n* = 10,004), only 43.1% of patients had acute coronary syndrome [24].

A prospective single-center study by Baran et al. involved 29.9% of STEMI patients [25]. This study showed no correlation of hemodynamics, left ventricular ejection fraction (LV EF), or laboratory parameters with the SCAI shock stage [25]. The Altshock-2 registry study, which included 43% of MI CS patients, also showed that patients had no statistically significant differences in echocardiographic characteristics depending on the SCAI shock stage [26]. 

In the National Cardiogenic Shock Initiative study, SCAI shock stages were applied retrospectively to 300 MI CS patients (within the first 24 h). This study undertook early invasive hemodynamic evaluation and used mechanical circulatory support prior to revascularization. Despite the earliest possible identification of patients with MI CS, many important hemodynamic and laboratory parameters in the database were similar between shock stages [27].

Our analysis, as well as the above studies, showed no statistically significant differences in most of the laboratory and echocardiographic parameters; yet, a similar trend was observed for most of the other parameters used to determine the severity and extent of cardiac injury. Thus, it can be assumed that mortality risk in MI CS patients depends on shock severity, which is not associated with MI characteristics.

In a retrospective registry by Hector Gonzalez-Pacheco et al., patients in SCAI shock stages C, D, and E were more likely to have lower LV EF and higher rates of mechanical complications [28]. However, the delay between symptom onset and hospital admission was greater in patients in late SCAI shock stages (C, D, and E) compared with patients in early shock stages (A and B), and patients in late SCAI shock stages (C, D and E) mostly received no reperfusion therapy [28]. 

**Figure 5:**
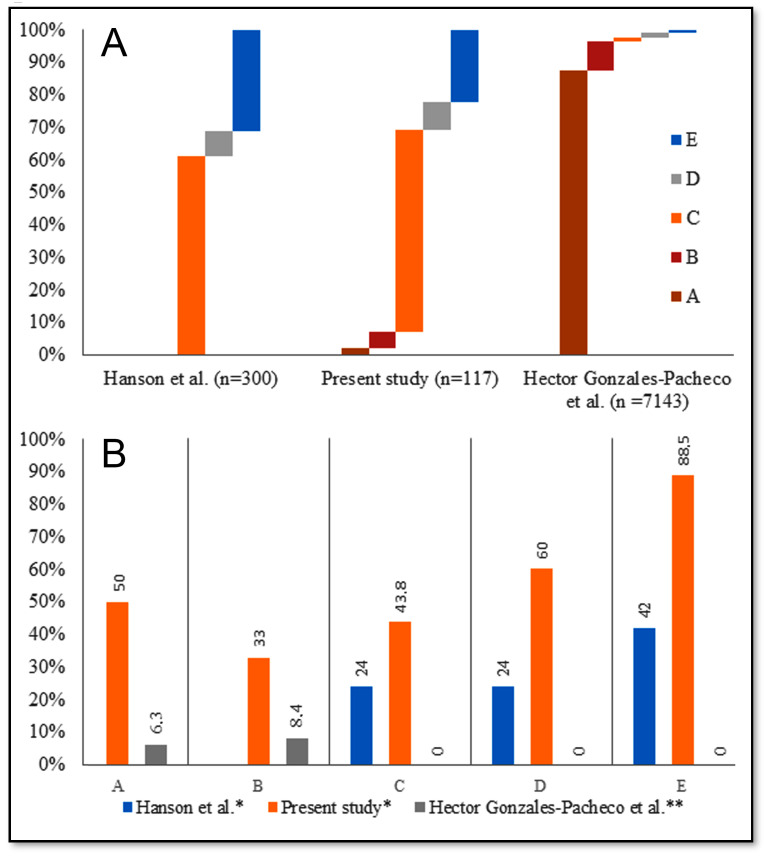
Distribution of SCAI SHOCK stages in each study [27,28]. Footnotes: (**A**) relative frequencies of shock stage classification, %; (**B**) short-term (in-hospital * or 30-day **) mortality in AMI-CS, %.

The existing data do not allow us to unequivocally reveal whether MI characteristics affect the SCAI shock severity. On the one hand, the above data may indicate the similarity of MI characteristics between stages [25,26,27], but on the other hand, the number of patients, the duration of data accumulation, and the characteristics of clinical databases in previous studies on MI CS differed significantly [27,28]. This resulted in a different shock distribution across the stages and affected the final result (Figure 5A). However, differences in sample characteristics did not affect an increasing trend in mortality, which was revealed in the studies (Figure 5B).

The results of the study by Hector Gonzalez-Pacheco et al. show that early reperfusion therapy is still inaccessible for reducing mortality from MI CS in low- and middle-income countries [28]. High mortality rates from MI CS reported in registries in Europe and America, which have advanced in this area, in addition to the results of our study, suggest a more complex nature of shock in this subgroup of patients and a number of unaccounted characteristics for shock outcome prediction [29,30]. One of these characteristics is the monocyte count in MI-CS patients upon admission (Supplementary Table S4), which is associated with the SCAI shock stage.

Cardiogenic shock is associated with systemic inflammation and multiorgan failure [31]; convincing data were obtained on the correlation between shock severity and elevated levels of highly sensitive C-reactive protein, interleukin-1b, interleukin-6, and tumor necrosis factor alpha [32]. The content of cytokines in the serum of MI CS patients was analyzed, and an indirect marker of the monocyte activity was used for prognostic purposes in a number of studies [33]. The peak monocyte count recorded during the immediate postinfarction period serves as a marker of the extent of cardiac injury and a prognostic factor for the course of postinfarction LV remodeling [34,35]. Yet, the role of monocytes in isolated cardiogenic shock remains poorly understood. At the same time, a biomarker based on the lymphocyte and monocyte count, called the lymphocyte-to-monocyte ratio (LMR), is an innovative biomarker of inflammation. A lower LMR upon admission is convincingly associated with an increased risk of in-hospital mortality in MI CS patients [34]. 

In our study, the lowest mean monocyte counts were observed in stages D and E (Figure 2). Catecholamines essential to these stages are known to indirectly increase the level of interleukin 10 (IL-10) [35]. IL-10 has a suppressive effect on both the innate and adaptive immune responses. This can lead to CD4+ T cell lymphopenia; however, no direct correlation with a low circulating monocyte count has been reported in the literature. At the same time, the latter occurs in patients with septic shock, since shock is known to induce monocyte apoptosis in response to decreased CD14 expression [36,37]. The concentration of IL-10 in patients with septic shock is associated with poor clinical outcomes [38]. Thus, the combination of cardiogenic and undiagnosed septic shock is a more likely hypothesis explaining the decreased monocyte count in stages D and E. In turn, the mixed nature of CS is the subject of studies currently being performed. The role of catecholamine-mediated IL-10 release in shock progression is unknown [39]. A further in-depth study of the effect of activation of the innate immune system on the course of MI CS will probably reveal the desired therapeutic and/or diagnostic targets that can change the course and prognosis of CS.

In addition, an analysis of predictor significance levels for the polynomial logistic regression model showed that a low pH (acidemia) was strongly associated with a higher likelihood of refractory shock (stage E) compared to other variables (Supplementary Table S4; Figure 3). This is probably due to the fact that severe systemic acidemia impairs the cardiovascular response to catecholamines. Thus, low pH predicts mortality, which should be taken into account regardless of shock severity. Previous studies have shown that blood pH decreases as the SCAI shock stage advances; therefore, the determination of reference pH values for all SCAI shock stages can contribute to the early and optimal stratification of MI CS patients.

The more reliable association with mortality for pH compared with lactate when both were included in the same multivariate model highlights the important role of the disruption of homeostatic mechanisms. The inclusion of pH in the current clinical guidelines for acute heart failure seems appropriate, given that at the moment, laboratory verification of shock is represented only by the measurement of lactate levels.

The revealed predictors of shock severity can supplement and improve the existing criteria for SCAI shock classification, which can enable the faster and more accurate stratification of MI CS patients depending on shock severity. In addition, further in-depth study of the effect of the revealed shock predictors on the course and prognosis of CS may contribute to our understanding of the nature of progression and severity of this MI complication.

### Study Limitations

A retrospective registry study has inherent limitations, such as missing data that could affect the findings. Shock staging was primarily based on clinical and laboratory findings, without the invasive measurement of hemodynamic parameters other than central venous pressure. There was no analysis of the group of patients with later development of cardiogenic shock due to a lack of data. We did not have baseline blood pressure data, so some patients with chronically low blood pressure could have been falsely classified as stage B. The number of patients in stages A and B was small. Therefore, the conclusions are not strong enough. Mortality was not adjusted for age. Also, the present study presents as a limitation the fact that most of the patients from our center underwent PCI, which certainly affected and improved the outcomes. The absence of PCI can probably serve as a predictor of a severe course of CS. In addition, this study was retrospective in nature; therefore, there are missing values in a number of measurements, which, unfortunately, is inevitable in retrospective data analysis.

## 5. Conclusions

Along with the current understanding of the correlation of severe tissue perfusion injuries and the worst prognosis with the SCAI shock stage in MI CS patients, we emphasize a high discriminatory potential of the pH level to predict refractory shock. The monocyte count upon admission can be a predictor of shock severity, but this requires further study as a marker of innate immune system activation. The characteristics of MI in different SCAI shock stages did not show significant differences and did not indicate shock severity. The reasons for the heterogeneity of MI CS with regard to the homogeneity of MI characteristics and the discovery of new predictors of shock severity are still disputable and require further investigation.

## Figures and Tables

**Table 1:** Adapted SCAI shock staging [21,22,23].

Stage	Characteristics
At risk (A)	Neither hypotension nor hypoperfusion Large, acute MI
Beginning (B)	Hypotension without hypoperfusion
Classic (C)	Hypoperfusion without deterioration
Deteriorating (D)	Hypoperfusion with deterioration Not RS
Extremis (E)	Hypoperfusion with deterioration and RS
Term	Definition
Large acute MI	HS TnI > 1 ng/mL
Hypotension/tachycardia	Presence of any of the following criteria: Admission SBP < 90 mmHg Minimum SBP < 90 mmHg during first 1 h Need for vasoactives to maintain SBP > 90 mmHg Admission MAP < 60 mmHg
Hypoperfusion	Presence of any following criteria: Admission lactate >2 mmol/L Urine output < 720 mL during first 24 h or <30 mL/h Cold, clammy skin Altered mental status
Deterioration	Presence of all following criteria: Number of vasoactives during first 1 h >1 and IABP during first 24 h Admission lactate > 2 mmol/L but <8 mmol/L
RS	Presence of any of the following criteria: Admission lactate >8 mmol/L pH < 7.2 CPR (A-modifier)

Footnotes: Abbreviation: CPR, cardiopulmonary resuscitation; HS TnI, Troponin-I, high sensitivity; IABP, intra-aortic balloon pump; MAP, mean artery pressure; MI, myocardial infarction; RS, refractory shock; SBP, systolic blood pressure.

**Table 2:** Clinical, laboratory, functional, anatomical features of patients with myocardial infarction complicated by cardiogenic shock according to SCAI scale classification (N = 117).

Parameter	N	A + B (*n* = 8)	C (*n* = 73)	D (*n* = 10)	E (*n* = 26)	*p* Value	Fisher’s Exact Test
Demographic data							
Age, years		65.5 (58; 75.5)	73 (66; 81)	80.5 (73.3; 82)	80 (78.3; 86.5)	0.009	
Male, *n* (%)	47	0 (0)	34 (46.6)	5 (50)	8 (30.8)	0.008	0.005
Female, *n* (%)	70	8 (100)	39 (53.4)	5 (50)	18 (69.2)		
Comorbidity							
Respiratory disease, *n* (%)	116	1 (12.5)	17 (23.6)	1 (10)	5 (19.2)	0.698	0.812
Urinary system diseases, *n* (%)	117	0 (0)	23 (31.5)	4 (40)	4 (15.4)	0.093	0.086
Gastrointestinal diseases, *n* (%)	117	6 (75)	50 (68.5)	8 (80)	13 (50.9)	0.229	0.257
Oncology, *n* (%)	115	0 (0)	4 (5.6)	0 (0)	0 (0)	0.463	0.785
Cerebrovascular disease, *n* (%)	116	2 (25)	11 (15.3)	0 (0)	4 (15.4)	0.487	0.5
CAD risk factors							
Smoking history, *n* (%)	79	4 (57.1)	21 (39.6)	1 (11.1)	1 (10)	0.07	0.073
Alcohol consumption, *n* (%)	84	1 (14.3)	5 (8.9)	0 (0)	0 (0)	0.489	0.554
Hypertension history, *n* (%)	117	7 (87.5)	69 (94.5)	9 (90)	24 (92.3)	0.849	
Diabetes, *n* (%)	117	1 (12.5)	25 (34.2)	5 (50)	3 (11.5)	0.185	0.083
Intensive care measures							
MV, *n* (%)	117	3 (37.5)	52 (71.2)	9 (90)	26 (100)	<0.001	<0.001
IABP, *n* (%)	110	0 (0)	7 (10.4)	10 (100)	10 (38.5)	<0.001	<0.001
Inotropes, *n* (%)	117	4 (50)	46 (63)	9 (90)	22 (84.6)	0.054	0.047
RRT, *n* (%)	109	0 (0)	7 (10.1)	1 (10)	3 (13)	0.8	0.945
Blood transfusion, *n* (%)	117	1 (12.5)	14 (19.2)	4 (40)	5 (19.2)	0.432	0.491
PCI, *n* (%)	111	5 (62.5)	49 (70)	8 (80)	14 (60.9)	0.903	0.806
Duration of intensive care measures							
MV duration, days	90	1 (1; 1)	3 (1; 7)	2 (2; 8)	1 (1; 4)	0.07	
ICU LOS, days	119	1.5 (1; 5)	5 (2; 13)	9.5 (4; 25.3)	1 (1; 5)	<0.001	
In-hospital LOS days	117	5 (1; 10.3)	10 (3; 16)	10.5 (5.3; 25.3)	1 (1; 5)	<0.001	
IABP duration, hours	24	NaN	61 (49; 61)	45 (30; 47)	129 (84; 664)	0.084	
Haemotransfusion, doses	24	NaN	2.9 (2; 3.5)	6.3 (1.8; 8.5)	2 (1; 2)	0.404	
Duration of RRT, min	7	NaN	3 (2.8; 3)	NaN	3 (2.5; 3.5)	0.693	
Clinical data							
GCS, score	113	15 (15; 15)	13 (10; 15)	15 (14; 15)	8 (6.8; 12)	<0.001	
SBP, mm Hg	114	96.5 (86.3; 131)	90 (76.5; 106)	91.5 (89.3; 94.8)	70 (60; 86)	<0.001	
Mean BP, mm Hga	115	72.2 (64.8; 93.8)	69 (53; 82)	71 (65; 85)	50 (44; 60)	0.001	
HR, beats per minute	116	87 (77; 100)	87 (65; 108)	101(94; 117)	99 (70; 116)	0.406	
RR, per minute	103	18 (17.8; 19.3)	18 (16; 22)	20 (17; 24)	18 (16; 20)	0.754	
CVP, mm Hg	83	7 (6; 8.25)	12 (9; 16)	12 (9; 13)	16 (10; 18)	0.062	
PHv	95	7.39 (7.36; 7.44)	7.3 (7.27; 7.34)	7.29 (7.26; 7.31)	7.14 (7.06; 7.18)	<0.001	
Laboratory (first 24 h)							
Lactate, mmol/L	104	1.7 (1.3; 1.7)	3.4 (2.4; 5.6)	4.8 (4.2; 6)	8.6 (6.9; 11.4)	<0.001	
Platelet, 10^3^/microL	117	222 (188; 263)	248 (190; 296)	202 (187.8; 269.8)	184 (139.3; 238)	0.032	
RBC count, 10^6^/microL	117	4.46 (4.1; 4.51)	4.38 (3.97; 5.05)	4.5 (4.28; 4.7)	4.19 (3.64; 4.9)	0.472	
Hemoglobin, g/dL	117	132 (119; 143)	133 (115;144)	129 (123.3; 135.8)	119 (103.3; 137.8)	0.32	
Hematocrit, %	117	0.37 (0.35;0.42)	0.39 (0.34; 0.43)	0.38 (0.35; 0.4)	0.36 (0.31; 0.43)	0.609	
WBC, 10^9^/microL	117	12.2 (10.7; 13.9)	13.7 (10.4; 16)	11.5 (10.1; 14.6)	13.7 (8.8; 17.6)	0.856	
Monocytes, 10^9^/microL	117	1.15 (0.96; 1.23)	0.98 (0.68; 1.32)	0.53 (0.44; 0.68)	0.78 (0.49: 0.94)	0.005	
Creatinine, mcmol/L	117	86.5 (76; 112)	131 (97; 166)	134.5 (104;172.5)	142.5 (116; 188)	0.028	
eGFR according to CKD-EPI, mL/min/1.73 m^2^	115	72 (56.3; 86)	39.5 (28.3; 56)	37 (28.5; 46.5)	35 (23; 47)	0.006	
Total protein, g/dL	74	64.5 (60.9; 69.4)	66 (59.4; 72)	65 (59.3; 68.8)	62.4 (53.9; 65)	0.417	
Glucose, mmol/L	116	7.73 (6.59; 8.87)	10.6 (8.5; 15.8)	11 (9.2; 14.5)	13.2 (8.2; 16.7)	0.235	
TBil, mcmoll/L	80	10.6 (6.88; 24.2)	14.2 (10; 21.7)	19 (13; 28.4)	16.2 (11.3; 43.4)	0.612	
Echocardiography at admission							
SV, ml	53	60 (43.5; 65)	41 (36; 52)	37 (31; 38)	41.5 (32; 52.3)	0.504	
MM, g/ml	42	267 (209; 288)	211 (180; 248)	NaN	204 (174.3; 272.8)	0.891	
MMI	42	142 (114; 143)	112 (99; 128)	NaN	112 (103; 151)	0.815	
IVC, mm	51	16 (15; 17)	20.5 (17.4; 22.1)	20 (17.8; 20)	19 (18; 22.5)	0.43	
LA, mL	53	81.2 (73.8; 88.6)	61.5 (43.5; 92)	41 (40; 47)	52.6 (43.3; 72)	0.344	
RA, mL	34	70.2 (59.3; 81)	59.7 (42; 88.3)	NaN	53 (40; 70)	0.604	
Mortality, *n* (%)	117	3 (37.5)	32 (43.8)	6 (60%)	23 (88.5)	0.002	<0.001
Risk scales							
ORBI, score	79	10.5 (8.5; 12.5)	17 (12; 18.3)	19 (15.5; 22.5)	19 (14; 22)	0.054	
ORBI, %	79	9 (5.2; 15.6)	35.4 (12.4; 45.6)	54.2 (28.3; 72.7)	47 (21.7; 70)	0.045	
SOFA, score (at admission)	27	5 (5; 5)	10 (6; 12.5)	10.5 (10; 11)	14.5 (11; 15)	0.129	
GRACE, %	117	7.5 (6; 16.3)	30 (12; 50)	29.5 (14; 53.3)	60 (40; 80)	<0.001	
CRUSADE, %	117	9.3 (6.5; 10.4)	13.6 (10.7; 19.5)	15.5 (9; 19.5)	19.5 (16.7; 19.5)	0.002	
GENEVA, score	116	4 (1; 5)	4 (1; 6)	6 (4.3; 6)	5 (1; 6)	0.136	
Dosage of vasopressors							
Dopamine dosage, mcg/kg/min	60	5 (4; 6)	5 (5; 10)	7 (3.5;8)	10 (6; 13.8)	0.113	
Epinephrine dosage, mcg/kg/min	11	NaN	0.05 (0.02; 0.1)	NaN	0.1 (0.1; 0.2)	0.146	
Nonepinephrine dosage, mcg/kg/min	51	0.05 (0.05; 0.05)	0.25 (0.18; 0.5)	0.3 (0.15; 0.6)	0.4 (0.25; 0.9)	0.095	
VIS at admission	84	5 (2.5; 6)	10 (5; 29)	23.5 (8.5; 46)	40 (10; 62)	0.023	
CPR prior to hospital arrival	116	0 (0)	3 (4.2)	0 (0)	4 (15.4)	0.106	0.140
Without CPR	116	5 (62.5)	45 (62.5)	9 (90)	11 (42.3)	0.106	0.140
CPR in hospital	116	3 (37.5)	24 (33.3)	1 (10)	11 (42.3)	0.106	0.140

Footnotes: Data are displayed as *n* (%) for categorical variables and median (interquartile range) for continuous variables. *p* values are for x2 test; Fisher’s exact test was used for small samples (categorical variables) and the Kruskal–Wallis test for continuous variables. Abbreviations: BP, blood pressure; AD, coronary artery disease; CI, cardiac index; CO, cardiac output; CPR, cardiopulmonary resuscitation; CVP, central venous pressure; EF, ejection fraction; eGFR, estimated glomerular filtration rate; GCS, Glasgow coma scale; HR, heart rate; IABP, intra-aortic balloon pump; ICU, intensive care unit; IVC, inferior vena cava; LA, left atrium artificial lung ventilation; MM, myocardial mass; LOS, length of stay; MV, mechanical lung ventilation; PCI, percutaneous coronary intervention; RA, right atrium; RBC, red blood cells; RR, respiratory rate; RRT, renal replacement therapy; SBP, systolic blood pressure; SV, stroke volume; TBil, total bilirubin; VIS, vasoactive inotropic score; WBC, white blood cells.

**Table 3:** The association between independent variables of MI and the SCAI shock stage in a polynomial logistic regression model.

Levels 2,3,4,5	Predictor				OR		95% CI			*p*
**2–5**	Constant				0.0		0.0–0.0			0.00
	MV: 1—yes, 2—no; 2–1				2.14 × 10^81^		2.67 × 10^78^–1.72 × 10^84^			0.00
	Lactate at the admission >2 mmol/L: 1—yes, 2—no				0.0		0.0–0.0			0.00
	Monocytes at the admission				6.63 × 10^16^		6.73 × 10^13^–6.54 × 10^19^			0.00
	SBP				0.51		0.24–1.06			0.07
	pHv				1.30 × 10^19^		1.72 × 10^14^–9.91 × 10^23^			1.64 × 10^−14^
	spO_2_/FioO_2_				0.67		0.57–0.79			1.81 × 10^−6^
	IABP—1, without IABP—2; 2–1				0.0		0.0–0.0			0.00
**3–5**	Constant				0.0		0.0–0.0			0.00
	MV: 1—yes, 2—no; 2–1				2.24 × 10^45^		2.79 × 10^42^–1.80 × 10^48^			0.00
	Lactate at the admission >2 mmol/L: 1—yes, 2—no				0.66		0.41–1.07			0.09
	Monocytes at the admission				34.39		1.65–716.91			0.02
	SBP				1.04		0.99–1.10			0.16
	pHv				2.45 × 10^7^		8.24 × 10^6^– 7.30 × 10^7^			0.00
	spO_2_/FioO_2_				1.01		0.99–1.02			0.31
	IABP—1, without IABP—2; 2–1				6.71		0.31–147.54			0.23
**4–5**	Constant				0.0		0.0–0.0			0.00
	MV: 1—yes, 2—no; 2–1				0.00		9.85 × 10^52^–9.85 × 10^52^			9.85 × 10^52^
	Lactate at the admission >2 mmol/L: 1—yes, 2—no				1.34		0.27–6.67			0.72
	Monocytes at admission				0.0		0.0–0.0			0.00
	SBP				1.33		1.16–1.52			4.35 × 10^−5^
	pHv				233.67		42.14–1295.62			4.35 × 10^−10^
	spO_2_/FioO_2_				0.98		0.95–1.00			0.76
	IABP—1, without IABP—2; 2–1				0.0		0.0–0.0			0.00
**Model Fit Measures**										
				**Overall Model Test**						
**Model**		**Deviance**	**AIC**	**R²N**		**χ²**		**df**	***p***	
1		36.59	84.58	0.78		102.28		21	1.1383 × 10^−12^	
Omnibus Likelihood Ratio Tests										
Predictor				χ²		Df		*p*		
MV: 1—yes, 2—no				5.60		3		0.13		
Lactate at the admission >2 mmol/L: 1—yes, 2—no				5.49		3		0.14		
Monocytes at admission				6.09		3		0.11		
SBP				6.245		3		0.10		
pHv				22.19		3		5.94 × 10^−5^		
IABP—1, without IABP—2				6.56		3		0.09		
spO_2_/FioO_2_				−5.24		3		1.00		

Footnotes: Data are displayed as *n* (%) for categorical variables and median (interquartile range) for continuous variables. *p* values are for the χ^2^” test; Fisher’s exact test was used for small samples (categorical variables) and the Kruskal–Wallis test for continuous variables. Abbreviations: IABP, intra-aortic balloon pump; MV, mechanical lung ventilation; SBP, systolic blood pressure. Abbreviations: IABP, intra-aortic balloon pump; MV, mechanical lung ventilation; SBP, systolic blood pressure.

## Data Availability

The datasets used and/or analyzed during the current study are available from the corresponding author on reasonable request.

## Supplementary Materials

The following supporting information can be downloaded at: https://www.mdpi.com/article/10.3390/jcm12247739/s1, Table S1: SCAI classification; Table S2. Characteristics of myocardial infarction; Table S3. Clinical, laboratory, functional, anatomical features versus SCAI stage of cardiogenic shock classification scale; Table S4. Model Coefficients—Levels SCAI: 2,3,4,5, reference level is 5.
