# Supplementary material for: SCAI Staging Application for Acute Myocardial Infarction-Related Cardiogenic Shock at a Single-Center Russian Registry
Source: J Clin Med. 2023 Dec 17;12(24):7739. doi: 10.3390/jcm12247739 (PMC10743544; doi:10.3390/jcm12247739)
Supplement: Supplementary file 1 [file jcm-12-07739-s001.zip › jcm-2700543-supplementary.pdf]

Supplementary

Table S1. SCAI classification.

Stage	Characteristics
At risk (A)	• Neither hypotension nor hypoperfusion<br>• Large acute myocardial infarction
Beginning (B)	• Hypotension without hypoperfusion
Classic (C)	• Hypoperfusion without deterioration
Deteriorating (D)	• Hypoperfusion with deterioration<br>• Not refractory shock
Extremis (E)	• Hypoperfusion with deterioration and refractory shock

Term	Definition
Large acute myocardial infarction	• HS TnI > 1 ng
Hypotension/tachycardia	Presence of any following criteria:<br>• Admission systolic BP <90mmHg<br>• Minimum systolic BP <90mmHg during first 1 h<br>• Need for vasoactives to maintain systolic BP >90 mmHg<br>• Admission MAP <60mmHg
Hypoperfusion	Presence of any following criteria:<br>• Admission lactate >2 mmol/L<br>• Urine output <720 ml during first 24 h or <30 ml/h<br>• Cold, clammy skin<br>• Altered mental status
Deterioration	Presence of all following criteria:<br>• Number of vasoactives during first 1 h > 1 and IABP during first 24 h<br>• Admission lactate >2 mmol/l but <8 mmol/L
Refractory shock	Presence of any following criteria:<br>• Admission lactate >8 mmol/L<br>• pH<7.2<br>• CPR (A-modifier)

Table S2. Characteristics of myocardial infarction.

Parameter	N	A and B (n=8)	C (n=73)	D (n=10)	E (n=26)	*P* value
CHARACTERISTICS OF MI						
STEMI	117	7 (87.5%)	58 (79.5 %)	8 (80.0 %)	18 (69.2 %)	0.641
non-STEMI		1 (12.5%)	15 (20.5 %)	2 (20.0 %)	8 (30.8 %)	
Primary MI	117	5 (62.5%)	39 (53.4 %)	5 (50.0 %)	20 (76.9 %)	0.190
Recurrent MI		3 (37.5%)	34 (46.6 %)	5 (50.0 %)	6 (23.1 %)	
CABG in anamnesis	105	0 (0.0%)	2 (3.0 %)	1 (14.3 %)	1 (4.0 %)	0.482
PCI in anamnesis	112	0 (0.0%)	9 (13.0 %)	1 (10.0 %)	3 (11.5 %)	0.781
Q-wave MI	116	7 (87.5%)	50 (68.5 %)	8 (80.0 %)	15 (60.0 %)	0.424

LOCALIZATION OF MI						
Anterior	117	5 (62.5%)	41 (56.2 %)	5 (50.0 %)	14 (53.8 %)	0.329
Inferior		3 (37.5%)	29 (39.7 %)	5 (50.0 %)	7 (26.9 %)	
Anterior-inferior		0 (0.0 %)	3 (4.1 %)	0 (0.0 %)	4 (15.4 %)	
RV MI		0 (0.0 %)	0 (0.0 %)	0 (0.0 %)	1 (3.8 %)	
MANAGEMENT						
Primary PCI	114	5 (62.5%)	45 (62.5 %)	9 (90.0 %)	13 (54.2 %)	0.711
Pharmaco-invasive strategy		2 (25.0%)	9 (12.5 %)	1 (10.0 %)	3 (12.5 %)	
Trombolysis only		0 (0.0 %)	2 (2.8 %)	0 (0.0 %)	1 (4.2 %)	
Conservative strategy		1 (12.5%)	16 (22.2 %)	0 (0.0 %)	7 (29.2 %)	
EXTENT OF CAD						
1-vessel	98	0 (0.0 %)	15 (23.4 %)	2 (20.0 %)	6 (33.4 %)	0.590
2-vessel		2 (33.3%)	20 (31.3 %)	4 (40.0 %)	4 (22.2 %)	
3-vessel		4 (66.7%)	29 (45.3 %)	4 (40.0 %)	8 (44.4 %)	
TIME						
"Door-to-balloon", min.	95	107.0 (51.8; 159.0)	62.5 (52.3; 138.0)	116.0 (59.8;228.5)	51.0 (43.0; 115.0)	0.324
"Of onset of symptoms to admission", min.	107	164.0 (95.0; 641.0)	218.5 (120.0;453.0)	306.0 (150.8;498.0)	153.0 (90.0; 410.0)	0.710
ECHOCARDIOGRAPHY ON ADMISSION						
EF LV, %	103	52.5 (42.0;62.3)	44.0 (31.0; 54.3)	42.0 (39.0; 47.3)	45.0 (40.0; 56.0)	0.729
ESV LV, mL	55	86.0 (61.5-93.0)	59.0 (37.0-76.5)	63.0 (40.5-73.5)	62.0 (45.0-98.0)	0.740
EDV LV, mL	78	120.0 (108.0;135.0)	103.0 (80.0; 130.0)	98.0 (94.0;124.0)	100.0 (75.0; 129.0)	0.592
PCWP, mm Hg	43	11.8 (11.7; 12.0)	13.6 (10.7; 16.6)	17.7 (15.3; 18.2)	14.8 (11.7; 18.2)	0.708
RV pressure, mmHg	48	27.0 (27.0; 38.0)	41.0 (35.5; 55.0)	30.0 (28.5; 35.0)	42.5 (36.3; 47.3)	0.191
CI, L/min/m2	51	2.15 (2.02; 2.27)	2.1 (1.6; 2.5)	1.9 (1.8; 2.3)	2.2 (1.7; 2.3)	0.994
IVLC	50	2.19 (1.69; 2.25)	1.6 (1.2; 2.1)	1.4 (1.2; 1.7)	1.7 (1.3; 2.0)	0.700
LABORATORY AT ADMISSION						
CK, mcg/L	117	645 (201.0;768.0)	175.0 (102.0;599.0)	380.5 (123.3;1422.8)	200.5 (100.5;413.3)	0.476
CK-MB, mcg/L	117	61.0 (45.8; 73.3)	46.0 (25.0; 74.0)	42.0 (26.8;331.0)	51.5 (35.8; 114.3)	0.529
Troponin at the admission, ng/mL	89	2.80 (0.715; 16.6)	0.33 (0.05; 2.29)	0.79 (0.61; 5.90)	0.37 (0.08; 0.72)	0.289
Lactate, mmol/L	104	1.70	3.4	4.8	8.6	< 0.001

		(1.30; 1.70)	(2.4; 5.6)	(4.2; 6.0)	(6.9; 11.4)	
PHv	95	7.39 (7.36; 7.44)	7.30 (7.27; 7.34)	7.29 (7.26; 7.31)	7.14 (7.06; 7.18)	< 0.001
Monocytes, 10^9/microL	117	1.15 (0.96; 1.23)	0.98 (0.68; 1.32)	0.53 (0.44; 0.68)	0.78 (0.49: 0.94)	0.005

Footnotes: Data displayed as n (%) for categorical variables and median (interquartile range) for continuous variables. P value is for x^2 test and Fisher's exact test was used for small samples (categorical variables) and Kruskal-Wallis test (continuous variables). Abbreviation: CABG, coronary artery bypass graft; CAD, coronary artery disease; CI, cardiac index; CO, cardiac output; CK, creatine kinase; CPK, creatine phosphokinase; EF, ejection fraction; EDV, end-diastolic volume; ESV, end-systolic volume; IVLC, index of violation of local contractility; LV, left ventricular; MI, myocardial infarction; PCWP, pulmonary capillary wedge pressure; PCI, percutaneous coronary intervention; RV, right ventricular; STEMI, ST elevation myocardial infarction.

Table S3. Clinical, laboratory, functional, anatomical features versus SCAI stage of cardiogenic shock classification scale.

Parameter	N	A+B (n=8)	C (n=73)	D (n=10)	E (n=26)	*P* value	Fisher's exact test
DEMOGRAPHIC DATA							
Age, years	119	65.5 (58.0; 75.5)	73.0 (66.0; 81.0)	80.5 (73.3; 82.0)	80.0 (78.3; 86.5)	0.009	
Male, n (%)	117	0 (0.0%)	34 (46.6 %)	5 (50.0 %)	8 (30.8 %)	0.008	0.005
Female, n (%)		8 (100 %)	39 (53.4 %)	5 (50.0 %)	18 (69.2 %)		
COMORBIDITY							
Respiratory disease, n (%)	116	1 (12.5 %)	17 (23.6 %)	1 (10 %)	5 (19.2 %)	0.698	0.812
Urinary system diseases, n (%)	117	0 (0.0 %)	23 (31.5 %)	4 (40.0 %)	4 (15.4 %)	0.093	0.086
Gastrointestinal diseases, n (%)	117	6 (75.0 %)	50 (68.5 %)	8 (80.0 %)	13 (50.9 %)	0.229	0.257
Oncology, n (%)	115	0 (0.0 %)	4 (5.6 %)	0 (0.0 %)	0 (0.0 %)	0.463	0.785
Cerebrovascular disease, n (%)	116	2 (25.0 %)	11 (15.3 %)	0 (0.0 %)	4 (15.4 %)	0.487	0.500
CAD RISK FACTORS							
Smoking history, n (%)	79	4 (57.1 %)	21 (39.6 %)	1 (11.1 %)	1 (10.0 %)	0.070	0.073
Alcohol consumption, n (%)	84	1 (14.3 %)	5 (8.9 %)	0 (0.0 %)	0 (0.0 %)	0.489	0.554
Hypertension history, n (%)	117	7 (87.5%)	69 (94.5%)	9 (90.0%)	24 (92.3%)	0.849	
Diabetes, n (%)	117	1 (12.5%)	25 (34.2 %)	5 (50.0 %)	3 (11.5 %)	0.185	0.083
INTENSIVE CARE MEASURES							
MV, n (%)	117	3 (37.5 %)	52 (71.2 %)	9 (90.0 %)	26 (100%)	< 0.001	< 0.001
IABP, n (%)	110	0 (0.0 %)	7 (10.4 %)	10 (100 %)	10 (38.5 %)	< 0.001	< 0.001

Inotropes, n (%)	117	4 (50.0 %)	46 (63.0 %)	9 (90.0 %)	22 (84.6 %)	0.054	0.047
RRT, n (%)	109	0 (0.0 %)	7 (10.1 %)	1 (10.0 %)	3 (13.0 %)	0.800	0.945
Blood transfusion, n (%)	117	1 (12.5 %)	14 (19.2 %)	4 (40.0 %)	5 (19.2 %)	0.432	0.491
PCI, n (%)	111	5 (62.5 %)	49 (70.0 %)	8 (80.0 %)	14 (60.9 %)	0.903	0.806
DURATION OF INTENSIVE CARE MEASURES							
MV duration, days	90	1 (1.0; 1.0)	3.0 (1.0; 7.0)	2.0 (2.0; 8.0)	1.0 (1.0; 4.0)	0.070	
ICU LOS, days	119	1.5 (1.0; 5.0)	5 (2.0; 13.0)	9.5 (4.0; 25.3)	1 (1.0; 5.0)	< 0.001	
In hospital LOS days	117	5.0 (1.0; 10.3)	10.0 (3.0; 16.0)	10.5 (5.3; 25.3)	1.0 (1.0; 5.0)	< 0.001	
IABP duration, hours.	24	NaN	61.0 (49.0; 61.0)	45.0 (30.0; 47.0)	129.0 (84.0; 664.0)	0.084	
Haemotransfusion, doses	24	NaN	2.9 (2.0; 3.5)	6.3 (1.8; 8.5)	2.0 (1.0; 2.0)	0.404	
Duration of RRT, min.	7	NaN	3.0 (2.8; 3.0)	NaN	3.0 (2.5; 3.5)	0.693	
CLINICAL DATA							
GCS, score	113	15.0 (15.0; 15.0)	13.0 (10.0; 15.0)	15.0 (14.0; 15.0)	8.0 (6.8; 12.0)	< 0.001	
SBP, mm Hg	114	96.5 (86.3; 131.0)	90.0 (76.5; 106.0)	91.5 (89.3; 94.8)	70.0 (60.0; 86.0)	< 0.001	
Mean BP, mm Hg[a]	115	72.2 (64.8; 93.8)	69.0 (53.0; 82.0)	71.0 (65.0; 85.0)	50.0 (44.0; 60.0)	0.001	
HR, beats per minute	116	87.0 (77.0; 100.0)	87.0 (65.0; 108.0)	101.0(94.0; 117.0)	99.0 (70.0; 116.0)	0.406	
RR, per minute	103	18.0 (17.8; 19.3)	18.0 (16.0; 22.0)	20.0 (17.0; 24.0)	18.0 (16.0; 20.0)	0.754	
CVP, mm Hg	83	7.0 (6.0; 8.25)	12.0 (9.0; 16.0)	12.0 (9.0; 13.0)	16.0 (10.0; 18.0)	0.062	
PHv	95	7.39 (7.36; 7.44)	7.30 (7.27; 7.34)	7.29 (7.26; 7.31)	7.14 (7.06; 7.18)	< 0.001	
LABORATORY (first 24 hours)							
Lactate, mmol/L	104	1.70 (1.30; 1.70)	3.4 (2.4; 5.6)	4.8 (4.2; 6.0)	8.6 (6.9; 11.4)	< 0.001	
Platelet, 10^3/microL	117	222.0 (188.0; 263.0)	248.0 (190.0; 296.0)	202.0 (187.8; 269.8)	184.0 (139.3; 238.0)	0.032	

RBC count, 10^6/microL	117	4.46 (4.10; 4.51)	4.38 (3.97; 5.05)	4.50 (4.28; 4.70)	4.19 (3.64; 4.90)	0.472	
Hemoglobin, g/dL	117	132.0 (119.0; 143.0)	133.0 (115.0;144.0)	129.0 (123.3; 135.8)	119.0 (103.3; 137.8)	0.320	
Hematocrit, %	117	0.37 (0.35;0.42)	0.39 (0.34; 0.43)	0.38 (0.35; 0.40)	0.36 (0.31; 0.43)	0.609	
WBC, 10^9/microL	117	12.2 (10.7; 13.9)	13.7 (10.4; 16.0)	11.5 (10.1; 14.6)	13.7 (8.8; 17.6)	0.856	
Monocytes, 10^9/microL	117	1.15 (0.96; 1.23)	0.98 (0.68; 1.32)	0.53 (0.44; 0.68)	0.78 (0.49: 0.94)	0.005	
Creatinine, mcmol/L	117	86.5 (76.0; 112.0)	131.0 (97.0; 166.0)	134.5 (104.0;172.5)	142.5 (116.0; 188.0)	0.028	
eGFR according to CKD-EPI, mL/min/1.73 m^2	115	72.0 (56.3; 86.0)	39.5(28.3; 56.0)	37.0 (28.5; 46.5)	35.0 (23.0; 47.0)	0.006	
Total protein, g/dL	74	64.5 (60.9; 69.4)	66.0 (59.4; 72.0)	65.0 (59.3; 68.8)	62.4 (53.9; 65.0)	0.417	
Glucose, mmol/L	116	7.73 (6.59; 8.87)	10.6 (8.5; 15.8)	11.0 (9.2; 14.5)	13.2 (8.2; 16.7)	0.235	
TBil, mcmoll/L	80	10.6 (6.88; 24.2)	14.2 (10.0; 21.7)	19.0 (13.0; 28.4)	16.2 (11.3; 43.4)	0.612	
ECHOCARDIOGRAPHY AT ADMISSION							
SV, mL	53	60.0(43.5; 65.0)	41.0 (36.0; 52.0)	37.0 (31.0; 38.0)	41.5 (32.0; 52.3)	0.504	
MM, g/mL	42	267.0 (209.0; 288.0)	211.0 (180.0; 248.0)	NaN	204.0 (174.3; 272.8)	0.891	
MMI	42	142.0 (114.0; 143.0)	112.0 (99.0; 128.0)	NaN	112.0 (103.0; 151.0)	0.815	
IVC, mm	51	16.0 (15.0; 17.0)	20.5 (17.4; 22.1)	20.0 (17.8; 20.0)	19.0 (18.0; 22.5)	0.430	
LA, mL	53	81.2 (73.8; 88.6)	61.5 (43.5; 92.0)	41.0 (40.0; 47.0)	52.6 (43.3; 72.0)	0.344	
RA, mL	34	70.2 (59.3; 81.0)	59.7 (42.0; 88.3)	NaN	53.0 (40.0; 70.0)	0.604	
Mortality, n (%)	117	3 (37.5%)	32 (43.8 %)	6 (60.0 %)	23 (88.5 %)	0.002	< 0.001
RISK SCALES							

ORBI, score	79	10.5 (8.50; 12.5)	17.0 (12.0; 18.3)	19.0 (15.5; 22.5)	19.0 (14.0; 22.0)	0.054
ORBI, %	79	9.0 (5.2; 15.6)	35.4 (12.4; 45.6)	54.2 (28.3; 72.7)	47.0 (21.7; 70.0)	0.045
SOFA, score (at admission)	27	5.0 (5.0; 5.0)	10.0 (6.0; 12.5)	10.5 (10.0; 11.0)	14.5 (11.0; 15.0)	0.129
GRACE, %	117	7.5 (6.0; 16.3)	30.0 (12.0; 50.0)	29.5 (14.0; 53.3)	60.0 (40.0; 80.0)	< 0.001
CRUSADE, %	117	9.3 (6.5; 10.4)	13.6 (10.7; 19.5)	15.5 (9.0; 19.5)	19.5 (16.7; 19.5)	0.002
GENEVA, score	116	4.0 (1.0; 5.0)	4 (1.0; 6.0)	6.0 (4.3; 6.0)	5.0 (1.0; 6.0)	0.136
DOSAGE OF VASOPRESSORS						
Dopamine dosage, mcg/kg/min	60	5.0 (4.0; 6.0)	5.0 (5.0; 10.0)	7.0 (3.5;8.0)	10 (6.0; 13.8)	0.113
Epinephrine dosage, mcg/kg/min	11	NaN	0.05 (0.02; 0.1)	NaN	0.1 (0.1; 0.2)	0.146
Nonepinephrine dosage, mcg/kg/min	51	0.05 (0.05; 0.05)	0.25 (0.18; 0.50)	0.3 (0.15; 0.60)	0.4 (0.25; 0.90)	0.095
VIS at admission	84	5.0 (2.5; 6.0)	10.0 (5.0; 29.0)	23.5 (8.5; 46.0)	40.0 (10.0; 62.0)	0.023

Footnotes: Data displayed as n (%) for categorical variables and median (interquartile range) for continuous variables. P value is for x^2 test and Fisher's exact test was used for small samples (categorical variables) and Kruskal-Wallis test (continuous variables). [a] Measured as DBP + 1/3(SBP-DBP). Abbreviation: BP, blood pressure; CABG, coronary artery bypass graft; CAD, coronary artery disease; CI, cardiac index; CO, cardiac output; CPK, creatine phosphokinase; CVP, central venous pressure; EF, ejection fraction; eGFR, estimated glomerular filtration rate; EDV, end-diastolic volume; ESV, end-systolic volume; GCS, Glasgow coma scale; HR, heart rate; IABP, intra-aortic balloon pump; ICU, intensive care unit; IVC, inferior vena cava; IVLC, index of violation of local contractility; LA, left atrium artificial lung ventilation; LV MMI, left ventricular myocardial mass index; LV, left ventricular; MI, myocardial infarction; MM, myocardial mass; LOS, length of stay; PCWP, pulmonary capillary wedge pressure; MV, mechanical lung ventilation; PCI, percutaneous coronary intervention; RA, right atrium; RBC, red blood cells; RR, respiratory rate; RRT, renal replacement therapy; RV, right ventricular; SBP, systolic blood pressure; STEMI, ST elevation myocardial infarction; SV, stroke volume; TBil, total bilirubin; VIS, vasoactive inotropic score; WBC, white blood cells.

Table S4. Model Coefficients—Levels SCAI: 2,3,4,5, reference level is 5.

**Model Coefficients – Levels SCAI: 2,3,4,5, reference level is 5**					
**Levels 2,3,4,5**	**Predictor**	**Estimate**	**SE**	**Z**	**P**
2 - 5	Constant	-272.79614	0.19151	-1424.47738	0.000000
	MV: 1- yes, 2- no; 2 – 1	61.90871	1.32726	46.64390	0.000000
	Lactate at the admission >2 mmol/L: 1-yes, 2-no	1.47884	3.24233	0.45610	0.648316
	Monocytes at the admission	1.80463	1.59316	1.13273	0.257326

	SBP	0.06002	0.05211	1.15190	0.249362
	pHv	35.73758	1.21156	29.49705	0.000000
	spO2/FioO2	-0.00315	0.01290	-0.24450	0.806845
	IABP - 1, without IABP – 2; 2 – 1	0.07414	6.45575	0.01148	0.990837
3 - 5	Constant	-134.19794	0.15711	-854.18008	0.000000
	MV: 1- yes, 2- no; 2 – 1	56.99527	1.32726	42.94193	0.000000
	Lactate at the admission >2 mmol/L: 1-yes, 2-no	-2.94693	1.58835	-1.85534	0.063548
	Monocytes at the admission	2.01875	0.95866	2.10579	0.035222
	SBP	0.03170	0.01782	1.77870	0.075289
	pHv	18.05461	0.41444	43.56366	0.000000
	spO2/FioO2	0.00959	0.00658	1.45734	0.145023
	IABP - 1, without IABP – 2; 2 – 1	1.73392	1.25690	1.37952	0.167735
4 - 5	Constant	-18.82212	0.09941	-189.33695	0.000000
	MV: 1- yes, 2- no; 2 – 1	83.71000	0.00000	52677889.26618	0.000000
	Lactate at the admission >2 mmol/L: 1-yes, 2-no	-24.68303	0.09941	-248.29351	0.000000
	Monocytes at the admission	-11.86619	0.09313	-127.41874	0.000000
	SBP	0.07393	0.05369	1.37704	0.168499
	pHv	6.06121	0.70977	8.53972	0.000000
	spO2/FioO2	0.00317	0.01048	0.30244	0.762313
	IABP - 1, without IABP – 2; 2 – 1	-41.58445	0.00000	-6168406.31	0.000000

Model Fit Measures

				Overall Model Test		
**Model**	**Deviance**	**AIC**	**R^2_N**	χ^2	df	P
1	36.58527	84.58527	0.78079	102.27894	21	1.1383e-12

Omnibus Likelihood Ratio Tests

Predictor	χ^2	Df	P
MV: 1- yes, 2- no	5.60384	3	0.132558
Lactate at the admission >2 mmol/L: 1-yes, 2-no	5.49434	3	0.138978
Monocytes at the admission	6.09025	3	0.107301
SBP	6.24653	3	0.100213
pHv	22.19635	3	5.9373e-5
IABP - 1, without IABP – 2	6.55931	3	0.087353
spO2/FioO2	-5.24009	3	1.000000

Abbreviation: SBP, systolic blood pressure; IABP, intra-aortic balloon pump; MV, mechanical ventilation.
